# Evolutionarily Ancient Association of the FoxJ1 Transcription Factor with the Motile Ciliogenic Program

**DOI:** 10.1371/journal.pgen.1003019

**Published:** 2012-11-08

**Authors:** Shubha Vij, Jochen C. Rink, Hao Kee Ho, Deepak Babu, Michael Eitel, Vijayashankaranarayanan Narasimhan, Varnesh Tiku, Jody Westbrook, Bernd Schierwater, Sudipto Roy

**Affiliations:** 1 Institute of Molecular and Cell Biology, Proteos, Singapore, Singapore; 2 Max Planck Institute of Molecular Cell Biology and Genetics, Dresden, Germany; 3 Department of Biological Sciences, National University of Singapore, Singapore, Singapore; 4 NUS Graduate School for Integrative Sciences and Engineering, National University of Singapore, Singapore, Singapore; 5 ITZ Division of Ecology and Evolution, Stiftung Tierärztliche Hochschule, Hannover, Germany; 6 Department of Molecular and Cell Biology and Center for Integrative Genomics, University of California Berkeley, Berkeley, California, United States of America; 7 School of Biological Sciences, Nanyang Technological University, Singapore, Singapore; Fred Hutchinson Cancer Research Center, United States of America

## Abstract

It is generally believed that the last eukaryotic common ancestor (LECA) was a unicellular organism with motile cilia. In the vertebrates, the winged-helix transcription factor FoxJ1 functions as the master regulator of motile cilia biogenesis. Despite the antiquity of cilia, their highly conserved structure, and their mechanism of motility, the evolution of the transcriptional program controlling ciliogenesis has remained incompletely understood. In particular, it is presently not known how the generation of motile cilia is programmed outside of the vertebrates, and whether and to what extent the FoxJ1-dependent regulation is conserved. We have performed a survey of numerous eukaryotic genomes and discovered that genes homologous to *foxJ1* are restricted only to organisms belonging to the unikont lineage. Using a mis-expression assay, we then obtained evidence of a conserved ability of FoxJ1 proteins from a number of diverse phyletic groups to activate the expression of a host of motile ciliary genes in zebrafish embryos. Conversely, we found that inactivation of a *foxJ1* gene in *Schmidtea mediterranea*, a platyhelminth (flatworm) that utilizes motile cilia for locomotion, led to a profound disruption in the differentiation of motile cilia. Together, all of these findings provide the first evolutionary perspective into the transcriptional control of motile ciliogenesis and allow us to propose a conserved FoxJ1-regulated mechanism for motile cilia biogenesis back to the origin of the metazoans.

## Author Summary

Cilia are microtubule-based, hair-like organelles that project from the surfaces of eukaryotic cells. Protists use motile cilia for locomotion as well as for sensory perception. In metazoans, motile cilia also function in fluid transport over epithelia, such as in the mammalian lungs. Most vertebrate and some invertebrate cell-types differentiate non-motile primary cilia, which function exclusively in sensory transduction. It is believed that primary cilia arose from motile cilia through the loss of the motility apparatus. Cilia are complex organelles: a large number of proteins are involved in their assembly and maintenance. FoxJ1, a forkhead-domain transcription factor, is the master regulator of motile ciliogenesis in vertebrates. It is not known to what extent this transcriptional control is conserved and how it may have evolved. Here, we document the existence of FoxJ1 orthologs in several eukaryotic groups besides the vertebrates. FoxJ1 proteins from three representative phyla—Placozoa, Platyhelminthes, and Echinodermata—were able to activate the expression of ciliary genes when mis-expressed in zebrafish embryos. Moreover, inactivation of FoxJ1 in planaria (Platyhelminthes) abolished motile cilia differentiation. These results provide new insights into the transcriptional regulation of motile cilia biogenesis outside the vertebrates and demonstrate a remarkable conservation of the activity of FoxJ1.

## Introduction

Cilia are primitive, cell surface-associated filamentous organelles with wide-spread distribution among the protozoans and most metazoan phyla. The hair-like extension of the cilium is called the axoneme - a structure that typically comprises nine peripheral microtubule doublets arranged around a central pair of singlet microtubules (the 9+2 arrangement) as in the motile cilia, or lacking the central pair (the 9+0 arrangement) as in the immotile primary or sensory cilia [1]. Besides these differences in microtubular organization, axonemes of motile cilia are decorated with dynein arms which confer motility through ATP-dependent sliding of the microtubule doublets relative to each other. The axoneme is enveloped by the ciliary membrane, an extension of the cell membrane, and at its base are the nine triplet microtubules of the basal body. Assembly and maintenance of the cilium from the complex set of constituting proteins depends upon a unique transport mechanism termed intraflagellar transport (IFT) [2]. This process involves the continual ferry of cargo proteins by IFT particles from the base to the tip of the axoneme, and back to the base. Cilia have varied functions, which seem to have evolved in synchrony with the strategic location of the organelle at the cell surface. These range from cellular locomotion, fluid transport and as platforms for signal transduction [3]. Given these diverse roles, defective cilia have been implicated in several human pathologies [3]–[5]. Consequently, the biogenesis as well as the functions of cilia in animal development and physiology is currently under intense scrutiny.

An outstanding question in the field of ciliary biology is how the expression of the complex ciliary proteome, likely comprising several hundreds of proteins, is regulated at the transcriptional level [6]. Some insight into this problem has come from the analyses of the **r**egulatory **f**actor **X** (Rfx) family of transcription factors. Rfx proteins of *Caenorhabditis elegans* and *Drosophila melanogaster* are essential for primary cilia formation [7]–[11]. In mice, the Rfx homologs Rfx3 and Rfx4 regulate genes required for the formation and function of primary as well as motile cilia [12]–[14]. Motile cilia and motile cilia-specific genes are completely absent from *C. elegans*. In *D. melanogaster*, only sperm cells elaborate motile cilia (flagella); however, a role for the Rfx factors in the regulation of flagellar synthesis has not been defined. Yet another transcription factor, FoxJ1, plays a critical role in the regulation of ciliary gene expression in several species of vertebrates [15]–[17]. Unlike the Rfx proteins, FoxJ1 seems to have a function exclusively in the control of motile, but not primary cilia formation. Moreover, evidence from the zebrafish as well as mice suggests that FoxJ1 functions upstream of the Rfx factors in the ciliogenic pathway [17], [18].

Although the cilium has a highly conserved structure and an ancient origin, the evolutionary history of the transcriptional regulation of ciliary genes has remained an unsolved problem. Recently, two studies, using an entirely *in silico* approach, have garnered evidence that the Rfx family of transcription factors and the ciliary genes evolved independently [19], [20]. The authors proposed that the ciliary genes were gradually “re-wired” to come under the regulation of the Rfx proteins. It is presently not known whether any aspect of the FoxJ1-dependent control of ciliogenesis is conserved outside of the vertebrates. This is a pertinent issue, because in contrast to Rfx, FoxJ1 activity is not just necessary, but strikingly, is also sufficient for programming cells to assemble functional motile cilia [15]–[17]. In addition, it is generally agreed that the origin of the motile cilium predated the immotile primary cilium, and that the latter derived from the former through the progressive loss of the motility machinery [21]. Furthermore, whereas FoxJ1 seems to have a dedicated role in the generation of cilia [16], [17], [22], [23], the Rfx proteins have been implicated in the regulation of genes linked to many other processes, besides ciliogenesis [12], [24]. Here, we report the identification of FoxJ1 homologs from many diverse phylogenetic groups. Using transient transgenesis, we found that mis-expression of the FoxJ1 proteins from three representative invertebrate phyla – Placozoa, Platyhelminthes and Echinodermata - in zebrafish embryos, led to the ectopic induction of a number of motile ciliary genes that we have previously established to be canonical targets of vertebrate FoxJ1. To complement this sufficiency function, we explored whether these invertebrate FoxJ1 proteins in their native species are involved in the ciliogenic pathway. Indeed, RNAi-mediated abolition of FoxJ1 activity in the flatworm *S. mediterranea* strongly impaired the differentiation of motile cilia. These data underscore a functional conservation in motile ciliary gene regulation by FoxJ1 transcription factors across diverse groups of metazoans.

## Results

### FoxJ1 proteins are present only in the fungi/metazoa group within the unikonts

In order to first gain insights into the phylogenetic distribution of FoxJ1 proteins, we searched a total of 215 organisms representing all of the major phylogenetic groups within the eukaryotes for the presence of FoxJ1. FoxJ1 is part of the large Fox family of transcription factors which are characterized by a distinct DNA-binding domain (the forkhead domain or FKH) spanning ∼100 amino acids. In the mammals (mice), Fox proteins have been classified into several subfamilies ranging from FoxA-FoxS [25]. We performed BLASTP and TBLASTN searches against chosen proteomes/genomes with the FKH domain of human FOXJ1, and the proteins retrieved with an E-value less than E-2 were considered to be potential orthologs. However, the FKH family members exhibit a high degree of conservation within the FKH domain, and a large number of proteins which belonged to FKH families other than FoxJ1 were obtained using this method. In order to filter this information and identify the true FoxJ1 orthologs, a two-pronged approach was employed. First, all the identified proteins were subjected to phylogenetic analyses by aligning their FKH domain sequences with those of the FKH domains for 42 Fox family members of the mouse [25]. Independently, a reverse BLAST was also done against the human (nr) protein database using the FKH domains of the proteins identified in the initial search. A total of 60 FoxJ1 proteins could be identified using a combination of the two approaches; of these, only 43 can be considered definite orthologs as they reliably grouped with mouse FoxJ1 (bootstrap (BS)>95) (Table 1).

**Table 1:** List of organisms with an identifiable FoxJ1 based on reverse BLAST and/or phylogenetic analyses.

S. No.	Supergroup	Group	Phylum/subphylum	Organism	E-value	Reverse BLAST	Annotation	Phylogeny (domain)	Bootstrap	Phylogeny (Full length)*	Bootstrap*
1	Opisthokonta	Animalia	Placozoa	*Trichoplax adhaerens*	8E-38	FoxJ1	Hypothetical protein TRIADDRAFT_17444	FoxJ1	99		
2	Opisthokonta	Animalia	Cnidaria	*Hydra magnipapillata*	9E-32	FoxJ1	Similar to FoxJ1	FoxJ1	49		
3	Opisthokonta	Animalia	Cnidaria	*Nematostella vectensis*	3E-39	FoxJ1	Predicted protein	FoxJ1	99		
4	Opisthokonta	Animalia	Annelida	*Capitella sp. I*	4E-47	FoxJ1	e_gw1.802.18.1	FoxJ1	99		
5	Opisthokonta	Animalia	Mollusca	*Lottia gigantea*	2E-44	FoxJ1	Fgenesh2_pg.C_sca_5000409	FoxJ1	99		
6	Opisthokonta	Animalia	Echinodermata	*Strongylocentrotus purpuratus*	1E-37	FoxJ1	FoxJ1	FoxJ1	100		
7	Opisthokonta	Animalia	Arthropoda	*Daphnia pulex*	9E-36	FoxJ1	Hypothetical protein	FoxJ1	97		
8	Opisthokonta	Animalia	Arthropoda	*Tribolium castaneum*	2E-32	FoxJ1	Hypothetical protein	FoxJ1	99		
9	Opisthokonta	Animalia	Arthropoda	*Pediculus humanus corporis*	1E-34	FoxJ1	Forkhead protein/forkhead protein domain, putative	FoxJ1	99		
10	Opisthokonta	Animalia	Chordata	*Branchiostoma floridae*	1E-37	FoxJ1	Hypothetical protein	FoxJ1	99		
11	Opisthokonta	Animalia	Chordata	*Danio rerio*	3E-50/1e-43	FoxJ1	FoxJ1a/FoxJ1b	FoxJ1	100 (J1a), 99 (J1b)		
12	Opisthokonta	Animalia	Chordata	*Tetraodon nigroviridis*	1E-47/6e-41	FoxJ1	Unnamed protein product	FoxJ1	99 (J1), 99 (J1.2)		
13	Opisthokonta	Animalia	Chordata	*Fugu rubripes*	1E-58/4.16E-51	FoxJ1	e_gw2.514.3.1/e_gw2.3.885.1	FoxJ1	100 (J1), 99 (J1.2)		
14	Opisthokonta	Animalia	Chordata	*Xenopus tropicalis*	5E-50/4e-41	FoxJ1	FoxJ1/FoxJ1.2	FoxJ1	100 (J1), 99 (J1.2)		
15	Opisthokonta	Animalia	Chordata	*Gallus gallus*	1E-49	FoxJ1	Similar to FoxJ1	FoxJ1	100		
16	Opisthokonta	Animalia	Chordata	*Homo sapiens*	5E-51	FoxJ1	FoxJ1	FoxJ1	100		
17	Opisthokonta	Animalia	Chordata	*Mus musculus*	3E-51	FoxJ1	FoxJ1	FoxJ1	100		
18	Opisthokonta	Animalia	Chordata	*Rattus norvegicus*	1E-51	FoxJ1	FoxJ1	FoxJ1	100		
19	Opisthokonta	Animalia	Chordata	*Bos taurus*	1E-51	FoxJ1	FoxJ1	FoxJ1	100		
20	Opisthokonta	Animalia	Chordata	*Canis familiaris*	1E-50	FoxJ1	Similar to FoxJ1	FoxJ1	100		
21	Opisthokonta	Animalia	Chordata	*Pongo pygmaeus*	3.6E-75	FoxJ1	FoxJ1	FoxJ1	100		
22	Opisthokonta	Animalia	Chordata	*Cavia porcellus*	2.8E-75	FoxJ1	FoxJ1	FoxJ1	100		
23	Opisthokonta	Animalia	Chordata	*Erinaceus europaeus*	4.3E-58	FoxJ1	FoxJ1	FoxJ1	100		
24	Opisthokonta	Animalia	Chordata	*Loxodonta africana*	3.6E-75	FoxJ1	FoxJ1	FoxJ1	100		
25	Opisthokonta	Animalia	Chordata	*Gorilla gorilla*	4.4E-75	FoxJ1	FoxJ1	FoxJ1	100		
26	Opisthokonta	Animalia	Chordata	*Microcebus murinus*	3.6E-68	FoxJ1	FoxJ1	FoxJ1	100		
27	Opisthokonta	Animalia	Chordata	*Myotis lucifugus*	4.7E-71	FoxJ1	FoxJ1	FoxJ1	100		
28	Opisthokonta	Animalia	Chordata	*Ochotona princeps*	2.4E-75	FoxJ1	FoxJ1	FoxJ1	100		
29	Opisthokonta	Animalia	Chordata	*Procavia capensis*	3E-74	FoxJ1	FoxJ1	FoxJ1	100		
30	Opisthokonta	Animalia	Chordata	*Pteropus vampyrus*	6.1E-70	FoxJ1	FoxJ1	FoxJ1	100		
31	Opisthokonta	Animalia	Chordata	*Tupaia belangeri*	1.5E-46	FoxJ1	FoxJ1	FoxJ1	100		
32	Opisthokonta	Animalia	Chordata	*Felis catus*	1.8E-59	FoxJ1	FoxJ1	FoxJ1	100		
33	Opisthokonta	Animalia	Chordata	*Equus caballus*	8E-52	FoxJ1	Similar to FoxJ1	FoxJ1	100		
34	Opisthokonta	Animalia	Chordata	*Macaca mulatta*	8E-52	FoxJ1	FoxJ1	FoxJ1	100		
35	Opisthokonta	Animalia	Chordata	*Tursiops truncatus*	2.5E-74	FoxJ1	FoxJ1	FoxJ1	100		
36	Opisthokonta	Animalia	Chordata	*Monodelphis domestica*	9E-52	FoxJ1	Similar to forkhead transcription factor HFH-4	FoxJ1	100		
37	Opisthokonta	Animalia	Chordata	*Ornithorhynchus anatinus*	6E-51	FoxJ1	Similar to forkhead transcription factor HFH-4	FoxJ1	100		
38	Opisthokonta	Animalia	Chordata	*Oryctolagus cuniculus*	1E-51	FoxJ1	FoxJ1	FoxJ1	100		
39	Opisthokonta	Animalia	Chordata	*Pan troglodytes*	2E-51	FoxJ1	FoxJ1	FoxJ1	100		
40	Opisthokonta	Animalia	Chordata	*Taeniopygia guttata*	3E-38	FoxJ1	Similar to FoxJ1	FoxJ1	99		
41	Opisthokonta	Fungi	Ascomycota	*Aspergillus clavatus*	8E-27	FoxJ1	Forkhead transcription factor Fkh1/2, putative	Sister to FoxJ1–J3, FoxK1–K2		Sister to FoxJ2 and FoxJ3	44
42	Opisthokonta	Fungi	Ascomycota	*Aspergillus niger*	1E-27	FoxJ1	Forkhead transcription factor Fkh1/2	Sister to FoxJ1–J3, FoxK1–K2		Sister to FoxJ2 and FoxJ3	60
43	Opisthokonta	Fungi	Ascomycota	*Candida glabrata*	1E-25	FoxJ1	Hypothetical protein	Sister to FoxK1–K2		Sister to FoxP1–P4, FoxR1–R2	51
44	Opisthokonta	Fungi	Ascomycota	*Eremothecium gossypii*	5E-26	FoxJ1	AER369Cp	FoxJ1	31	Foxj1	66
45	Opisthokonta	Fungi	Ascomycota	*Saccharomyces cerevisiae*	2E-24	FoxJ1	Forkhead protein	Sister to FoxK1–K2		Foxj1	48
46	Opisthokonta	Fungi	Ascomycota	*Schizosaccharomyces pombe*	7E-25	FoxD2	Fork head transcription factor Fhl1	Foxj1	52	Unresolved	
47	Opisthokonta	fungi	Ascomycota	*Aspergillus oryzae*	3E-27	FoxJ1	Forkhead transcription factor Fkh1/2	Sister to FoxJ1–J3, FoxK1–K2		Sister to FoxJ2 and FoxJ3	47
48	Opisthokonta	fungi	Ascomycota	*Aspergillus terreus*	6E-27	FoxJ1	Forkhead box protein C2	Sister to FoxJ1–J3, FoxK1–K2		Sister to FoxJ2 and FoxJ3	46
49	Opisthokonta	Fungi	Ascomycota	*Coccidioides immitis*	2E-25	FoxJ1	Hypothetical protein CIMG_03727	FoxJ1	32	Sister to FoxJ2 and FoxJ3	47
50	Opisthokonta	Fungi	Ascomycota	*Coccidioides posadasii*	3E-25	FoxJ1	Fork head domain containing protein	FoxJ1	32	Foxj1	53
51	Opisthokonta	Fungi	Ascomycota	*Schizosaccharomyces japonicus*	3E-23	FoxJ1	Fork head transcription factor 1	Sister to FoxO1, O3, O4, O6		Sister to FoxP1–P4, FoxR1–R2	59
52	Opisthokonta	Fungi	Ascomycota	*Stagonospora nodorum*	9E-23	FoxJ1	Hypothetical protein SNOG_11882	Sister to FoxJ1–J3, FoxK1–K2		Unresolved	
53	Opisthokonta	Fungi	Basidiomycota	*Laccaria bicolor*	4E-15	FoxJ1	Predicted protein	Sister to FoxP1–P4		Foxh1	52
54	Opisthokonta	Fungi	Microsporidia	*Encephalitozoon cuniculi*	2E-26	FoxJ1	Transcription factor (forkhead domain)	Sister to FoxJ1–J3, FoxK1–K2		Sister to FoxJ2 and FoxJ3	39
55	Opisthokonta	Fungi	Blastocladiomycota	*Allomyces macrogynus*	4.01858E-25	FoxJ1	forkhead box J3	sister to FoxJ2–J3		Sister to FoxP1–P4, FoxR1–R2	97
56	Opisthokonta	Choanozoa	Choanozoa	*Monosiga brevicollis*	2.95E-29	FoxJ1	fgenesh2_pg.scaffold_5000329	sister to FoxJ1–J3; FoxK1–K2			

* Only shown for Fungal Fox proteins.

FoxJ1 homologs could not be identified from any of the bikonts (Archaeplastida, Excavata, Rhizaria and Chromalveolata); intriguingly, this includes *Chlamydomonas reinhardtii*, an organism where the biology of the prototypical 9+2 flagellum has been best studied. Rfx factors are also absent from this algal protist, implying that despite the high degree of similarity between its flagella and the motile cilia of metazoans, the transcriptional regulation of the biogenesis of these organelles is fundamentally different. Within the unikonts, FoxJ1 was not recovered from any organism of the amoebozoan lineage, including Acanthopodia, Archamoebae and Mycetozoa. Within the opisthokonts, true FoxJ1 orthologs are absent from Choanozoa and Nematoda (Table 1, Table S1), whereas FoxJ1 is present in many phyla such as Placozoa, Cnidaria, Annelida, Mollusca, Arthropoda, Echinodermata and Chordata (Table 1). Among the arthropods that we sampled, we found FoxJ1 in *Tribolium castaneum*, *Pediculus humanus* and *Daphnia pulex*, but not among species of the model genus *Drosophila*. As discussed earlier, like *C. elegans*, fruit flies are devoid of motile cilia except for the flagella that differentiate on their sperm cells. These flagella are peculiar in that they are synthesized in the cytoplasm without the involvement of IFT, and as our data show, or FoxJ1. In addition to the FKH domain, we also used the full length sequence of human FOXJ1 to search for potential homologs. The difference in the results between these two strategies centered on 13 organisms which are representatives from the Fungi and few metazoan phyla (Chordata and Arthropoda); in these, a homolog could be identified only using full-length human FOXJ1. Conversely, in 6 organisms representing the Fungi, a homolog was identifiable using the FKH domain but not with the full-length sequence. All of the additional FoxJ1 sequences that were recovered by the full-length search belonged to the Fungi/Metazoa, and as with the domain-based analysis, no homolog was identified outside this group (Table 1, Table S2).

In contrast to the metazoan FoxJ1 proteins, those identified from few fungal species, along with FoxJ1 from *Monosiga brevicollis* (Choanozoa), yielded inconsistent data on phylogenetic analyses as well as reverse BLAST (Table 1) – in fact, only 3 fungal species, all belonging to Ascomycota - *Eremothecium gossypii*, *Coccidioides immitis* and *Coccidioides posadasii* had a FoxJ1 which was identifiable using both reverse BLAST and phylogeny. However, the bootstrap for all three was less than 50% (Table 1). A previous study identified few fungal proteins as FoxJ1 orthologs since those particular sequences grouped together in the same clade as the *Nematostella*, *Amphimedon* (Porifera) as well as the bilaterian FoxJ1 proteins [26]. It is worth noting though that the particular clade had a weak bootstrap support in all three trees used (NJ<50; ML<50; PP: 0.56). In addition, although the relationship between the Fox family members are well established, a number of studies have shown discrepant data in the grouping together of certain subfamilies such as FoxR1–FoxR2, FoxL1–FoxL2, FoxJ1 with FoxJ2–FoxJ3 and FoxN1–FoxN4 with FoxN2–FoxN3 [25]. Therefore, in order to resolve the correct identity of the fungal Fox proteins identified in our search, the full length sequences of the putative FoxJ1 homologs from the 15 fungal species which showed the presence of FoxJ1 either through reverse BLAST or phylogeny (low BS support) were each aligned with full-length mouse Fox protein family members. This strategy, together with reverse BLAST and phylogeny based solely on the FKH domain yielded four species - *E. gossypii*, *Saccharomyces cerevisiae*, *C. immitis* and *C. posadasii* with identifiable FoxJ1 using at least two of the methods (although BS values were less even in the analyses based on the full length sequence (Table 1)). These findings establish the presence of FoxJ1 homologs only in the unikonts, similar to the other family of ciliogenic transcription factors, Rfx. FoxJ1, however, has a more restricted distribution, and unlike Rfx is absent from the amoebozoan lineage, as well as from the Choanozoa and the Nematoda within the opisthokonts.

### FoxJ1 proteins from Placozoa sp. H4 and *Strongylocentrotus purpuratus* can activate motile ciliary genes in the zebrafish embryo

**Figure 1:**
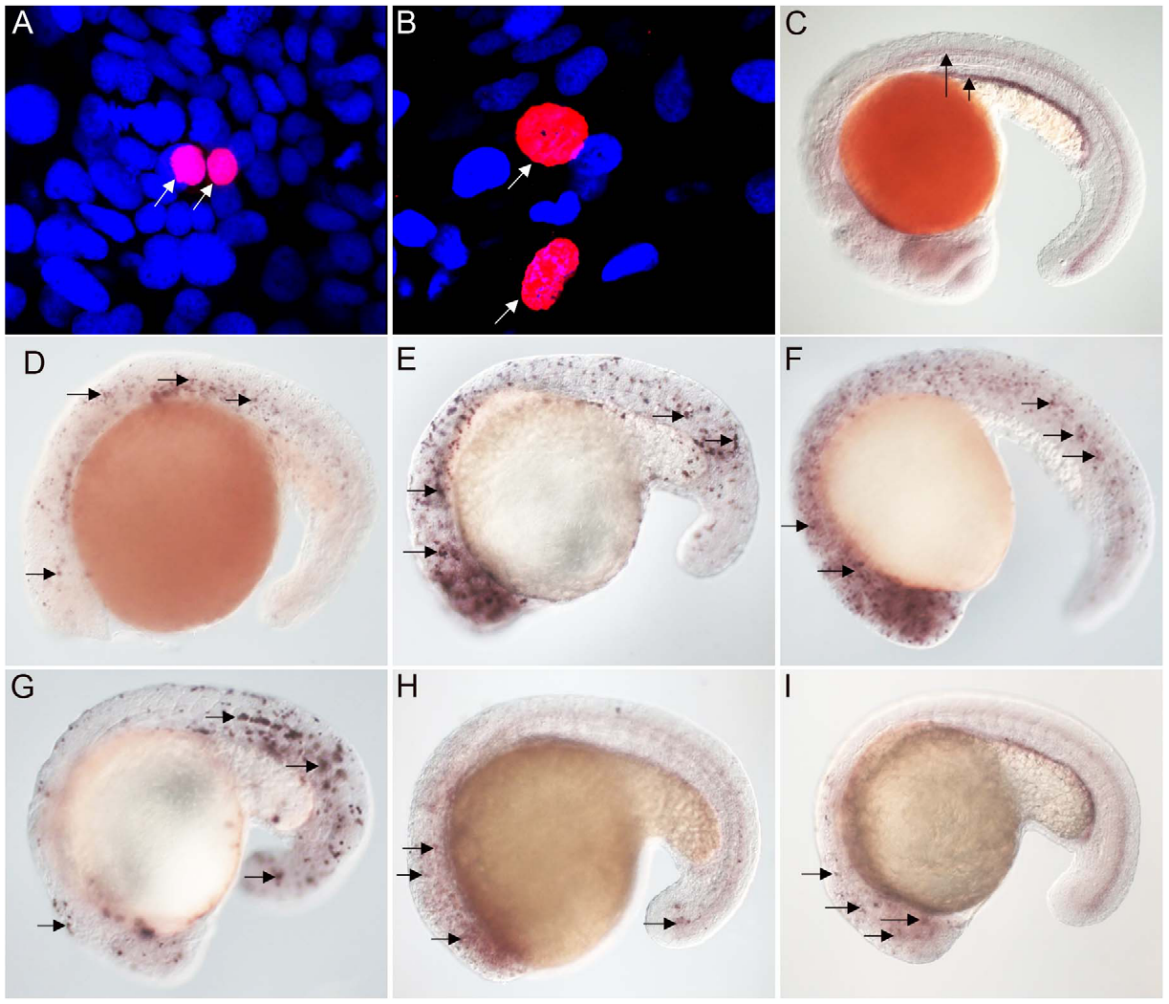
FoxJ1 from *T. adhaerens* and *S. purpuratus* are nuclear localized and can regulate the expression of ciliary genes. Anti-myc antibodies were used to detect Placozoa (A) and sea urchin (B) FoxJ1 (red, white arrow). Nuclei were stained with DAPI (blue). (C) Expression of *dynein intermediate chain* in the spinal cord (long arrow) and pronephric (kidney) duct (short arrow) of a wild-type zebrafish embryo. The *wdr78* and *efhc1* genes are expressed in a similar pattern in wild-type embryos (see Figure 2A and data not shown). Ectopic expression of *dynein intermediate chain* in embryos ectopically expressing placozoan (D) and sea urchin (E) FoxJ1, respectively. Ectopic expression of *wdr78* in embryos ectopically expressing placozoan (F) and sea urchin (G) FoxJ1, respectively. Ectopic expression of *efhc1* in embryos ectopically expressing placozoan (H) and sea urchin (I) FoxJ1, respectively. Mis-expression of the different ciliary genes in D–I is indicated by the arrows. Embryos depicted are at 20 hpf, oriented anterior to the left, dorsal to the top.

Studies with vertebrate FoxJ1 have established its role as a master regulator of motile ciliogenesis, meaning that the activity of the protein is both necessary as well as adequate for the generation of motile cilia [15]–[17]. Nothing is currently known about the biology of the non-vertebrate FoxJ1 proteins. To begin to investigate whether the role of FoxJ1 in regulating motile ciliogenesis is generally conserved, we first performed a mis-expression assay. For this, we selected two FoxJ1 proteins, from the placozoan species, Placozoa sp. H4 and the echinoderm *S. purpuratus*, as representatives, and then evaluated their transcriptional activity through transgenic expression in zebrafish embryos. Using this strategy, we have earlier shown that mis-expression of zebrafish FoxJ1 can ectopically activate a battery of motile ciliary genes [17]. The placozoans are an interesting model from an evolutionary standpoint since they are thought to represent the basal state of the metazoans [27]–[29]. However, other genome-level and phylogenomic analyses have instead placed the sponges as the most basal metazoan group [30], [31]. Regardless of the lack of a consensus view on this issue, anatomically the Placozoa are the simplest extant metazoans, with an elementary body plan and presence of only four cell-types, one of which bears motile cilia [28]. On the other hand, the echinoderms (and their sister phylum, the hemichordates), are the closest known relatives of the chordates. They typically reproduce through larval forms that have motile cilia, and hence also are an interesting group to incorporate in this study. Both the placozoan and *S. purpuratus* FoxJ1 proteins are 74% identical to human FoxJ1 in the FKH domain. Heat-inducible myc epitope tagged transgenic constructs for the two genes, i.e., *hs::myc-Pl-foxJ1* (Placozoa) and *hs::myc-Sp-foxJ1* (*S. purpuratus*), were made and expressed in zebrafish embryos using transient transgenesis as described previously for zebrafish FoxJ1 [17]. FoxJ1 proteins from both species localized to the nuclei of zebrafish cells (Figure 1A–1B).

**Figure 2:**
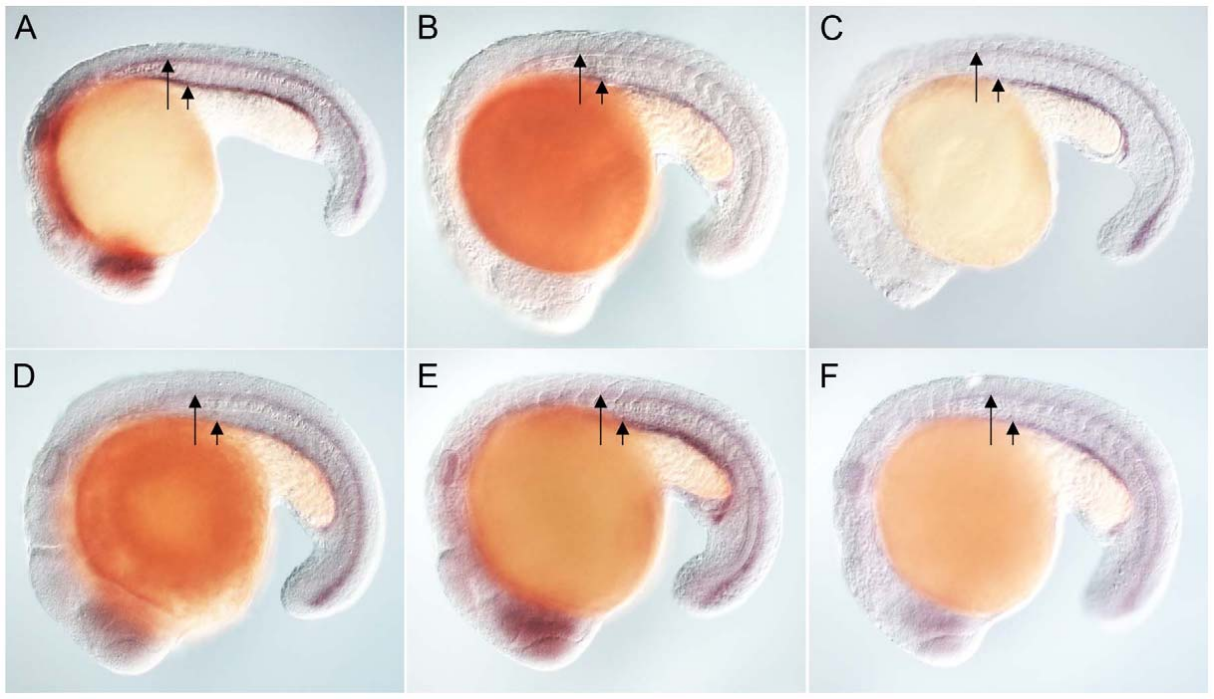
Zebrafish FoxJ2 and FoxJ3 are unable to induce the expression of ciliary genes. (A) Expression of *efhc1* in the spinal cord (long arrow) and pronephric (kidney) duct (short arrow) of a wild-type zebrafish embryo, and in embryos ectopically expressing zebrafish FoxJ2 (B) and FoxJ3 (C), respectively. (D) Expression of *spag6* in the spinal cord (long arrow) and pronephric (kidney) duct (short arrow) of a wild-type zebrafish embryo, and in embryos ectopically expressing zebrafish FoxJ2 (E) and FoxJ3 (F), respectively. Embryos depicted are at 20 hpf, oriented anterior to the left, dorsal to the top.

For exploring the transcriptional activity of the placozoan and echinoderm FoxJ1, we selected five well-established zebrafish FoxJ1 targets: *efhc1*, *spag6*, *wdr78*, *tektin1* and *dynein intermediate chain* that encode motile cilia-specific components and are hyper-induced in response to ectopic expression of zebrafish FoxJ1 [17]. Remarkably, both the placozoan and the *S. purpuratus* FoxJ1 proteins robustly induced all 5 ciliary genes (Figure 1C–1I, data not shown). Induction of these genes was lineage-independent, and could be observed in cells which under normal circumstances do not form motile cilia, indicating the sufficiency of the placozoan and echinoderm FoxJ1 proteins to activate a battery of motile ciliary genes in a wide diversity of zebrafish cell types, just like zebrafish FoxJ1 [17]. The Fox family members share a high degree of conservation in their FKH domain. For instance, mouse FoxJ1 is 56% identical in the FKH domain to the closely related FoxJ2, and 47% identical to the distantly related FoxN3 proteins. Although the different Fox family members are known to control distinct developmental and physiological processes through the regulation of discrete sets of target genes, the high degree of conservation in the DNA binding FKH domain raises the possibility that over-expression of the proteins could inappropriately lead to cross-activation of targets owing to the commonality in their DNA recognition motif. Hence, induction of the zebrafish motile ciliary genes by the placozoan and echinoderm FoxJ1 proteins could merely be reflective of such an effect. To negate this possibility, we cloned zebrafish *foxJ2* and *foxJ3*, *fox* genes that are most closely related to *foxJ1* [25], and mis-expressed them in zebrafish embryos using the heat-inducible transient transgenesis method described above. Unlike FoxJ1, neither FoxJ2 nor FoxJ3 was able to ectopically induce the expression of any of the ciliary genes (Figure 2A–2F). Besides the DNA binding FKH domain, a number of transcriptional activating motifs have been reported in FoxJ1. These include four regions rich in acidic amino acids (A1, A2, A3 and A4), a winged helix transcriptional activation region II motif and a proline-rich region [32], [33]. Alignment of mouse FoxJ1 and FoxJ2 sequences showed limited similarity in the acidic region A3, proline-rich region and the region II motif, but no conservation in the remaining three acidic regions (A1, A2, A4). A similar comparison with the FoxJ3 protein revealed limited homology only in the acidic region A4 and the proline-rich region, but no conservation among the remaining motifs important for transcriptional activation. Thus, a significant degree of divergence in the transcriptional regulatory domains of closely related Fox family members could explain their abilities to regulate distinct sets of target genes.

### Inactivation of a FoxJ1 homolog impairs motile cilia differentiation in *S. mediterranea*

**Figure 3:**
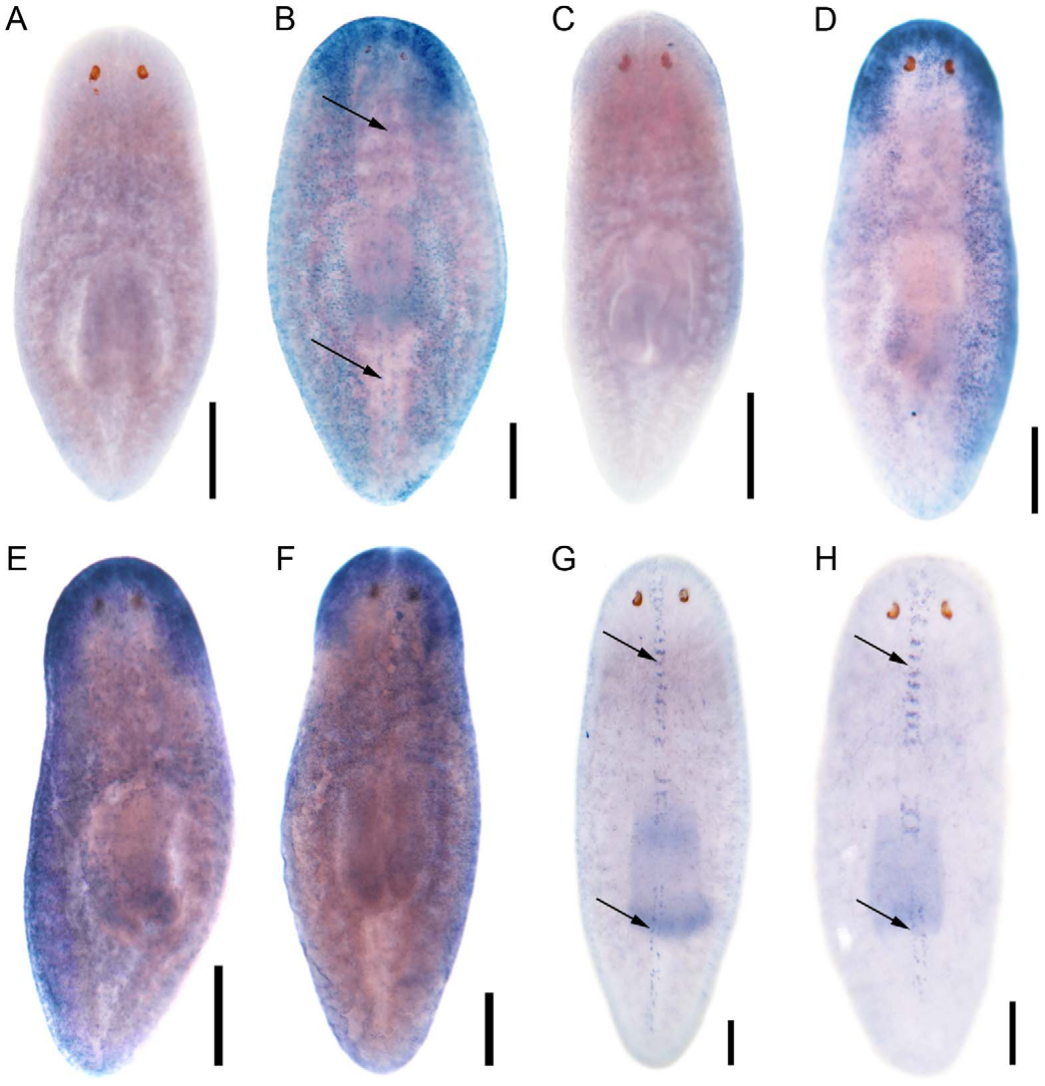
*S. mediterranea foxJ1* genes are expressed in ciliated tissues. Sense control (A) and expression pattern of *Smed-foxJ1-4* depicted in dorsal (B) and ventral view (E). Sense control (C) and expression pattern of *Smed-ift172* shown in dorsal (D) and ventral view (F). Expression pattern of *Smed-foxJ1-1* (G) and expression pattern of *Smed-foxJ1-2* (H). Arrows in B, G and H denote expression in the dorsal stripe of presumptive ciliated sensory cells. Scale bars: 300 µm for A–F and 200 µm for G–H.

**Figure 4:**
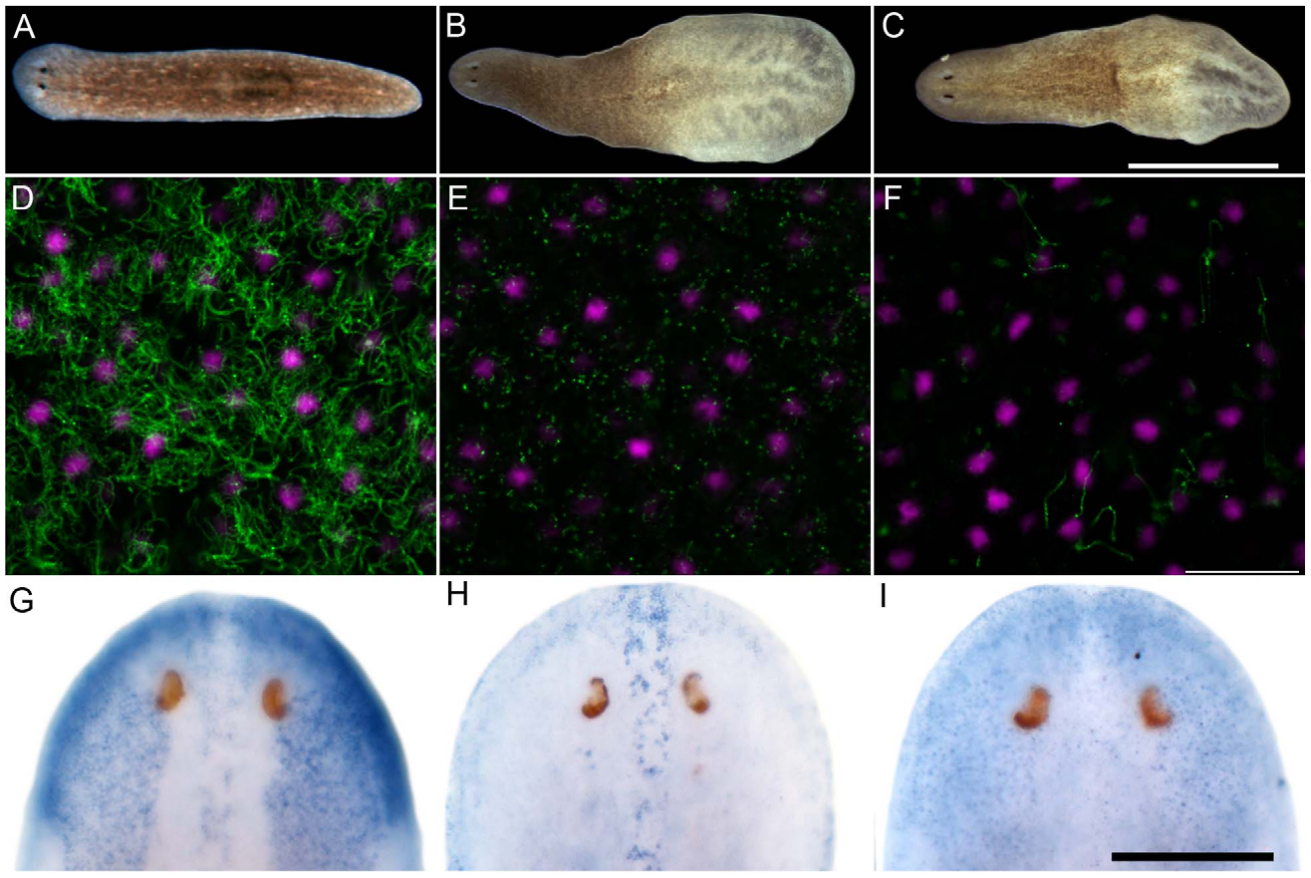
*S. mediterranea foxJ1-4* is required for the differentiation of motile cilia. (A–C) Control (A), *ift172*(RNAi) (B) and *foxJ1-4*(RNAi) worms (C) 14 days after the last RNAi feeding. Worms shown in panels B and C display tissue edema. Scale bar: 1 mm. (D–F) Anti-α-tubulin staining (green) of the multi-ciliated ventral epithelium in control (D), *ift172*(RNAi) (E) and *foxJ1-4*(RNAi) worms (F), respectively. The even spacing of nuclei (magenta) characteristic of the ventral epithelium demonstrates epithelial integrity in E and F. Images are single optical sections. Scale bar: 20 µm. (G) *foxJ1-4* expression is unaffected by control RNAi (*dsred*). (H) *foxJ1-1* expression is not altered in a *foxJ1-4*(RNAi) worm. (I) *foxJ1-4* expression is substantially reduced in a *foxJ1-4*(RNAi) worm. Scale bar: 200 µm.

Encouraged by the mis-expression studies in the zebrafish embryo, we wished to gather evidence that the non-vertebrate FoxJ1 proteins indeed control motile cilia differentiation in the context of their native species, just like their vertebrate counterparts. Among the non-vertebrate *foxJ1* genes, spatio-temporal expression pattern of only the sea urchin homolog has been described previously. The gene is transcribed in larval cells that bear tufts of motile cilia, implicating a role in ciliogenesis [34]. However, the lack of well-established methods of gene manipulation in echinoderm larvae or placozoans, and the absence of FoxJ1 from the traditional genetically amenable invertebrate models, *C. elegans* and *D. melanogaster*, made us resort to the planarian *S. mediterranea*. *S. mediterranea* is a representative of the basal worm phylum Platyhelminthes (flatworms), characterized by unsegmented and acoelomic morphology. *S. mediterranea* is popular in regeneration research, and RNAi mediated loss of gene activity can be efficiently achieved in this organism. Much less appreciated is the fact that the ventral epithelium of the worms consists of multiciliated cells very similar to the mammalian respiratory epithelium [35]. Metachronal waves of ciliary beating propel the worm around on a film of mucus, making the system an ideal model for mammalian pulmonary mucociliary clearance. To determine whether these morphological similarities extended even to the transcriptional regulation of ciliogenesis, we first searched the genome of *S. mediterranea* for *foxJ1* homologs. Many genes occur in multiple copies in this species, and BLAST searches with human FoxJ1 yielded four separate sequences that phylogenetic analysis confirmed to be paralogous *S. mediterranea* FoxJ1 proteins (SMED-FOXJ1-1 through -FOXJ1-4; BS support for FOXJ1-4 was the highest (99.9%) while that for FOXJ1-1, FOXJ1-2, FOXJ1-3 was 87.4%, 58.1% and 96.4%, respectively). *In situ* hybridization showed that *Smed-foxJ1-4* is widely expressed in the ventral motile ciliated cells, and dorsally in the head and in a stripe along the midline (Figure 3B, 3E). The dorsal expression regions also correspond to ciliated cells, but the function of these cilia is presently unclear (motile or sensory). Overall, *foxJ1*-*4* is expressed in a manner highly reminiscent of core ciliary genes like *Smed-ift72*, which encodes a highly conserved component of the IFT machinery (Figure 3D, 3F). By contrast, the expression of *foxJ1-1* and *foxJ1-2* is much more limited, and is confined to the mid-dorsal stripe of presumptive ciliated sensory cells (Figure 3G, 3H). We failed to observe a distinct expression pattern for *foxJ1-3*. In order to determine whether any of the 4 *foxJ1* orthologs might function in planarian ciliogenesis, we targeted the genes individually and in combination by feeding worms with dsRNA. *ift172*(RNAi) served as positive control for cilia phenotypes [36]. Animals that received *ift172* RNAi gradually lost their gliding ability - instead, they moved by peristaltic waves of whole body contractions (“inchworming”, Video S1). Like the *ift172*(RNAi) animals, those with *foxJ1-4* (but not *foxJ1-1*, *-2* or *-3*) RNAi also lost their gliding ability and moved around by inchworming (Video S1). Furthermore, *ift172*(RNAi) and some *foxJ1-4*(RNAi) worms developed posterior edema (Figure 4A–4C). Both inchworming and edema formation have previously been described as hallmarks of cilia defects in planarians [36], [37], and thus, strongly indicated a function of FOXJ1-4 in planarian ciliogenesis. Direct visualization of ciliary morphology confirmed this notion. Anti-α-tubulin staining of the ciliary axonemes revealed a dense lawn of cilia on the ventral epithelium of control worms (Figure 4D). *ift172*(RNAi) worms showed drastically shortened cilia remnants (Figure 4E) [36], whereas the *foxJ1-4*(RNAi) animals were almost entirely devoid of cilia (Figure 4F). To ensure that the RNAi procedure specifically knocked down the targeted genes, we performed *in situ* hybridization on RNAi fed worms. Control RNAi with a sequence not present in the planarian genome (DsRed) had no effect on *foxJ1-4* expression (Figure 4G), nor did *foxJ1-4*(RNAi) affect *foxJ1-1* expression (Figure 4H). *foxJ1-4*(RNAi), however, substantially reduced the expression levels of *foxJ1-4* (Figure 4I). Using the mis-expression assay described for the placozoan and echinoderm FoxJ1 proteins in the foregoing section, we also found that SMED-FOXJ1-4 could robustly activate motile ciliary genes in zebrafish embryos (data not shown). These results demonstrate that just like the vertebrates, FoxJ1 is a critical rate-limiting component of motile cilia differentiation even in the phylogenetically distant planarians.

## Discussion

A large number of transcription factors important for bilaterian development seem to have evolved even before the bilaterians and cnidarians diverged, as the two groups show considerable similarity in transcription factor family size and diversity. Similarly, the placozoans also have a comparable number of transcription factor families; for example, 16 out of the 22 bilaterian Fox subfamilies could be identified in *T. adhaerens*. In comparison, sponges have a more restricted representation, thus, indicating that the expansion and diversification in the suite of transcription factors was an early event in animal evolution [26], [38]. The FKH domain containing proteins have been identified only among the opisthokonts [38]. Within the Bilateria, there are FoxA-FoxS subfamilies, of which FoxL, J, N and Q have been further subdivided. Amongst these, FoxR and FoxS are specific to the vertebrates, while another subfamily, FoxAB, appears to be invertebrate-specific. These together comprise the 22 bilaterian subfamilies of Fox proteins. Most species have lost one or more subfamily members except *Branchiostoma floridae,* the only organism as yet sequenced whose genome encodes all of the Fox family representatives [38]. In our study too, we were unable to identify suitable FoxJ1 homologs in Archaeplastida (plants and their relatives), Excavata, Rhizaria, and Chromalveolata. Amongst the unikonts, FoxJ1 could be identified only within the opisthokont lineage. All ciliated organisms outside the unikont lineage, and even within the unikonts some species of ciliated fungi such as *Batrachochytrium dendrobatidis* lack FoxJ1. Conversely, FoxJ1 is present in several fungal species that are known to lack cilia.

A survey of the Rfx family of transcription factors have shown that, similar to our findings with FoxJ1, they are absent from the bikonts, but are present in *Acanthamoeba castellani*, a basal group within the unikont lineage, although two domains characteristic of the Rfx proteins – the dimerization domain and domain C are missing. Furthermore, compared to FoxJ1, the Rfx proteins are more wide-spread within the opisthokonts [19], [20]. Two Rfx genes have been identified in the choanoflagellate *M. brevicollis* [20], but we were unable to find a genuine FoxJ1 homolog. This is in contrast to an earlier study that reported FoxJ1 in *M. brevicollis* along with several other Fox proteins (FoxJ2/3, FoxN1/N4), and classified all these Fox family members as primitive since these were the only Fox proteins identified in *M. brevicollis* [38]. Another analysis identified seven Fox genes in the *M. brevicollis* genome, of which only *FoxN1/4* and *FoxJ2* were described to be true orthologs. The remaining five genes show similarity to *FoxJ1/FoxJ2* on BLAST search, but their sequences are highly divergent, and hence they could not be classified into specific Fox subfamilies based on phylogeny [26]. In our present search, a *M. brevicollis* Fox protein was identified on BLAST using the human FOXJ1 FKH domain sequence, but it could not be classified as FoxJ1 based on phylogenetic analysis (Table 1). This concurs with a recent work that also failed to identify FoxJ1 from *M. brevicollis* [39]. On the other hand, reverse BLAST classifies the *M. brevicollis* protein as FoxJ1. The same results were obtained using the full-length human FOXJ1 sequence for identification of orthologs. In view of these conflicting lines of evidence, it seems parsimonious to state that the presence of a *bona fide* FoxJ1 protein in *M. brevicollis* remains controversial. It also emerges from the previous surveys of the Rfx factors [19], [20] and our current study of FoxJ1, that although these transcription factors have a checkered distribution, the core ciliary gene repertoire that they regulate is quite conserved across distant phyla, ranging from the protists all the way to humans. This implies that like the Rfx family members, FoxJ1 arose within the unikont lineage only after the evolution of cilia.

Using a mis-expression assay in zebrafish embryos, we have established that FoxJ1 proteins from three divergent taxonomic groups, the placozoans, platyhelminths and the echinoderms, have a conserved role in the regulation of motile ciliary genes. Despite their distant phyletic relationships, all three proteins could robustly activate the five canonical vertebrate FoxJ1 targets that we tested, whereas zebrafish FoxJ2 and FoxJ3, the two Fox proteins most closely related to zebrafish FoxJ1, failed to do so. Mis-expression of zebrafish, *Xenopus* and chicken FoxJ1 proteins in zebrafish, *Xenopus* and chicken embryos, respectively, not only activates motile ciliary genes, but also induces ectopic motile cilia biogenesis [15]–[17]. However, none of the invertebrate FoxJ1 proteins that we tested were sufficient for ectopic motile cilia formation in zebrafish embryos, indicating their incapability to fully institute the zebrafish motile ciliogenic pathway (data not shown). To rule out differences in the amounts of over-expressed FoxJ1 proteins as a cause of this discrepancy, we quantified their levels using Western blot. Although the placozoan FoxJ1 consistently showed lower levels of expression compared to the zebrafish and the sea urchin proteins (possibly due to disparity in codon usage between the placozoans and vertebrates or instability of the protein when expressed in a heterologous system), the latter two were expressed at more or less equivalent levels (Figure S1). In light of this finding, we argue that species-specific differences in the FoxJ1 DNA binding domains and their corresponding DNA recognition sequence, together with species-specific diversity in the repertoire of target genes are more plausible reasons for the inability of the invertebrate FoxJ1 proteins to induce ciliogenesis in zebrafish.

To uncover a requirement of FoxJ1 function in motile cilia differentiation outside of the vertebrates, we have complemented the mis-expression analysis with a loss-of-function study of the planarian homologs of FoxJ1. Out of four paralogous *foxJ1* genes in *S. mediterranea*, we found that one of them, *foxJ1-4*, is absolutely necessary for ciliogenesis. Similar to the loss of motile cilia in FoxJ1 deficient zebrafish, frogs and mice, *foxJ1-4*(RNAi) planarians lost the ciliation of their ventral epithelium, where *foxJ1-4* is expressed. The fact that some *foxJ1-4*(RNAi) animals additionally developed edema indicates that *foxJ1-4* is required for ciliogenesis also in the planarian excretory system, which consists of heavily ciliated protonephridial tubules [37], just like the pronephric ducts of zebrafish embryos [40]. Although two distinct *foxJ1* paralogs have been reported from *Xenopus* and zebrafish [17], [41], [42], [43], interestingly, planarians are unique amongst the species examined here, where we detected four *foxJ1*-homologues. *foxJ1* is not the first example of gene amplification in planarians. *noggins* and *noggin*-like genes have been amplified to a total of ten family members in planarians [44]; other examples include six *wnt11* homologues [45], and two β*-catenin* genes [46] apparently segregating signaling and cell adhesion function. In the zebrafish and frogs, the two *foxJ1* paralogs have distinct as well as overlapping expression patterns, and functional analysis of the zebrafish genes have underscored their unique as well as redundant roles in regulating ciliogenesis in the different motile cilia bearing tissues that express them [17], [41], [42], [43]. The expression patterns of 3 of the 4 different planarian *foxJ1* genes suggests that like the duplicated *foxJ1* genes of fishes and amphibians, some degree of sub-functionalization within the ancestral ciliogenic role has also occurred here, with the different paralogs being delegated to the regulation of ciliogenesis in distinct tissues. Whereas *foxJ1*-*4* is critically required for the differentiation of the motile multiple cilia on ventral epidermal cells, the *foxJ1-1* and *-2* genes are not. Instead, they could have a role in the formation of cilia in the dorsal midline.

In conclusion, our findings have uncovered an ancient link between FoxJ1 and the motile ciliogenic program, and provide evidence that this regulatory mechanism is an ancestral feature of metazoan evolution. The distribution of FoxJ1 across various phyletic groups seems to suggest that the transcription factor evolved in the context of multicellularity, at a time when sophisticated regulatory controls were required for the differentiation of cilia. In the zebrafish and mammals, FoxJ1 and Rfx have been shown to cross-regulate each other's expression in the ciliogenic pathway [17], [47]. It is now apparent from the earlier studies with Rfx and our current work with FoxJ1 that the two ciliogenic transcription factors coexist in several other species*;* to what extent they collaborate to control ciliogenesis in each of these instances remains to be determined. Finally, in organisms that lack FoxJ1 but bear motile cilia, ciliary differentiation could be programmed entirely by Rfx, or by yet undiscovered transcriptional mechanisms.

## Materials and Methods

### Taxa sampling to identify FoxJ1 proteins

The FKH domain of human FoxJ1 was used to search for potential orthologs in 215 species representing all the major eukaryotic lineages by conducting BLASTP and TBLASTN searches in various databases including the NCBI non-redundant protein sequences database (http://blast.ncbi.nlm.nih.gov/Blast.cgi), Joint Genomes Institute, Department of Energy (genome.jgi-psf.org), Human Genome Sequencing Center, Baylor College of Medicine (http://www.hgsc.bcm.tmc.edu), Broad Institute (www.broadinstitute.org), Ensembl Genome Browser (http://www.ensembl.org) and *Saccharomyces* Genome Database (www.yeastgenome.org). Only those organisms whose proteins gave an E-value less than E-2 were considered to have a potential FoxJ1 ortholog. The FKH domain of each protein identified in BLAST was extracted using SMART (http://smart.embl-heidelberg.de) database and aligned (ClustalX multiple sequence alignment program) [48] with the 42 mouse FKH domain sequences. These data were used for making a neighbor joining tree, which was viewed using Treeview [49] to ascertain the identity of each protein. Alternatively, and independent of the phylogenetic analyses, the FKH domain of each identified protein was also used for reverse BLAST search against the *Homo sapiens* non-redundant protein database (NCBI) in order to assign the identified proteins to a particular subclass. The above described BLAST as well as phylogenetic analyses was also done using the full-length human FoxJ1 sequence. In instances where the full-length FoxJ1 sequence could not be retrieved for a particular species, the longest available sequence was used for the analyses.

### Zebrafish strains

Wild-type zebrafish were maintained under standard conditions of fish husbandry. All experiments with zebrafish embryos were approved by the Singapore National Advisory Committee on Laboratory Animal Research.

### *foxJ1, foxJ2*, and *foxJ3* full-length cDNAs

Incomplete sequence and annotation details were available for the *Trichoplax adhaerens foxJ1* gene in the public repositories (genome.jgi.doe.gov). Therefore, a full-length clone (1578 bp) (JX569795) was derived from sequencing of a Placozoa sp. H4 cDNA library [50], [51]. The full-length *S. purpuratus foxJ1* (1407 bp) was amplified from a sea urchin larval cDNA pool based on the annotation available in NCBI. The full length zebrafish *foxJ2* (1551 bp) and *foxJ3* (1779 bp) were amplified from 1 day old zebrafish embryonic cDNA pool. Cloning of zebrafish *foxJ1a* was reported previously [17]. Planarian *foxJ1* homologs were cloned from cDNA obtained from an 8-day regeneration series as described previously [52].

### RNAi in *S. mediterranea* via dsRNA feeding

Gene silencing in *S. mediterranea* was performed as previously described [52]. Bacterial pellet resulting from 70 ml of culture was mixed with 150 µl liver paste (3 parts liver∶1 part water). For double RNAi experiments, 35 ml of each IPTG-induced culture was mixed prior to pelleting. Worms received three feedings of RNAi food (2 days in between) and were fixed for analysis 14 days after the last feed.

### Ectopic *foxJ1, foxJ2*, and *foxJ3* expression in zebrafish embryos

For generating the heat inducible placozoan, platyhelminth, sea urchin and zebrafish *foxJ1* as well as zebrafish *foxJ2* and *foxJ3* constructs, six myc epitope tags were amplified from the pCS2+MT vector and cloned into the *Xba*I-*Spe*I sites of the pHspIG heat-shock vector. Coding sequences of the different fox genes were then cloned downstream of the myc tag to generate the *hs::myc-Pl-foxJ1, hs::myc-Sm-foxJ1-4*, *hs::myc-Sp-foxJ1, hs::myc-Dr-foxJ1, hs::myc-Dr-foxJ2* and *hs::myc-Dr-foxJ3* transgenes, respectively. For assessing the ability of the different Fox proteins to activate the expression of zebrafish motile ciliary genes, zebrafish embryos injected with the different heat inducible *fox* gene constructs were heat shocked at 37°C for 1 h at 14 hours post-fertilization (hpf). Following this, the embryos were allowed to grow until 20 hpf at 28°C before fixation for *in situ* hybridization.

### Microinjections into zebrafish eggs

The different heat inducible transgene containing plamids were linearized and injected at a concentration of ∼25 ng/µl into freshly fertilized zebrafish eggs at the one-cell stage. The typical volume for injections was ∼1 nl.

### RNA *in situ* hybridization and antibody staining

For mRNA *in situ* hybridization, worms were euthanized, fixed, hybridized, and developed as previously described [53]. For antibody labeling of cilia, animals were euthanized in 5% weight/volume N-acetyl cysteine in PBS under gentle rocking for 5 min and fixed for 2 h at 4°C in Carnoy's fixative (6 parts ethanol, 3 parts chloroform, 1 part glacial acetic acid). Following intensive rinsing with methanol, animals were bleached overnight in 6% H_2_O_2_ in 80% methanol, rehydrated and stained with anti-α-tubulin antibodies (Sigma), and visualized with Alexa-fluor-555 conjugated anti-mouse secondary antibodies (Invitrogen). Whole mount *in situ* hybridization and antibody staining of zebrafish embryos were done according to standard protocols. Antisense mRNA probes were used for the following genes: *spag6*, *wdr78*, *efhc1*, *tektin1* and *dynein intermediate chain.* The following antibodies were used: mAb anti-acetylated tubulin (Sigma) and anti-c-Myc (A-14 (sc-789); Santa Cruz Biotechnology). Alexa-fluor-conjugated secondary antibodies (Molecular Probes) were used for detection of signals. Nuclei were stained with DAPI.

### Microscopy

Stained *S. mediterranea* preparations were mounted in 80% glycerol and imaged on a Zeiss LSM700 confocal microscope equipped with multi-emersion objectives. Stained zebrafish embryos were mounted in 70% glycerol. A Zeiss compound microscope (Axio Imager Z1) fitted with a Zeiss digital camera (AxioCam HRc Rev 2) and an Olympus Fluoview laser scanning confocal microscope were used for imaging.

## Supporting Information

Figure S1 Expression levels of invertebrate FoxJ1 proteins in zebrafish embryos. Western blot showing roughly equivalent levels of the zebrafish and sea urchin FoxJ1 proteins, but lower levels of expression of placozoan FoxJ1. Tubulin levels were measured as loading control.
(JPG)

Table S1 List of organisms in which FoxJ1 could not be identified either by reverse BLAST or phylogenetic analyses.
(XLSX)

Table S2 List of organisms in which FoxJ1 orthologs were searched using the full-length human FoxJ1 protein sequence.
(XLSX)

Video S1 Normal motility in wild-type and *foxJ1-1*(RNAi) worms compared to the inchworming gait observed in worms with *foxJ1-4*(RNAi) and *ift172*(RNAi).
(MOV)
