# Supplementary figures and images for: Evolutionarily Ancient Association of the FoxJ1 Transcription Factor with the Motile Ciliogenic Program
Source: PLoS Genet. 2012 Nov 8;8(11):e1003019. doi: 10.1371/journal.pgen.1003019 (PMC3493443; doi:10.1371/journal.pgen.1003019)

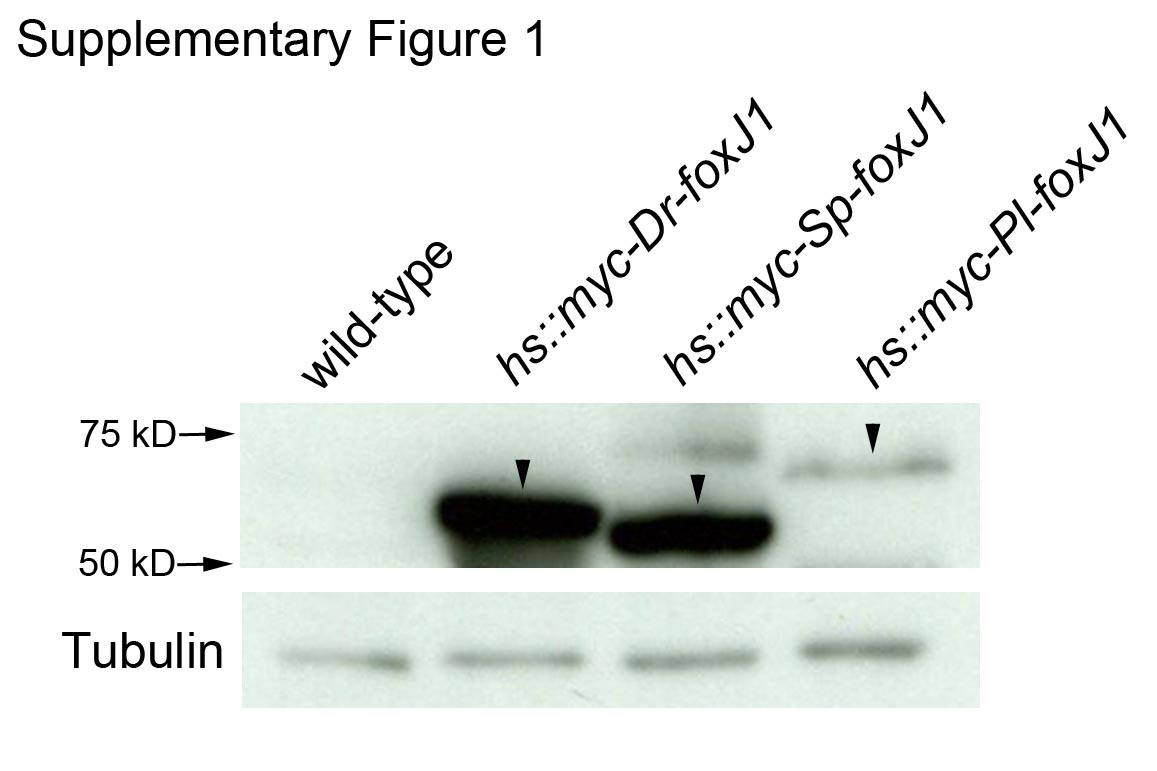

Supplement: Figure S1 — Expression levels of invertebrate FoxJ1 proteins in zebrafish embryos. Western blot showing roughly equivalent levels of the zebrafish and sea urchin FoxJ1 proteins, but lower levels of expression of placozoan FoxJ1. Tubulin levels were measured as loading control. (JPG) [file pgen.1003019.s001.jpg]
